# Supplementary material for: The tumour microenvironment shapes dendritic cell plasticity in a human organotypic melanoma culture
Source: Nat Commun. 2020 Jun 2;11:2749. doi: 10.1038/s41467-020-16583-0 (PMC7265463; doi:10.1038/s41467-020-16583-0)
Supplement: Supplementary file 1 — Supplementary Information [file 41467_2020_16583_MOESM1_ESM.pdf]

Supplementary Information

The tumour microenvironment shapes dendritic cell plasticity in a human organotypic melanoma culture

Di Blasio S et al.

Supplementary Table 1. Flow cytometry antibodies

Marker	Clone	Isotype	Fluorochrome	Cat#	Supplier	Dilution
CD1c	F10/21A3	Mouse IgG1, κ	BV421	565050	BD Biosciences	1:25 (1uL in 25uL)
CD14	MϕP9	Mouse IgG2b, κ	APCH7	560180	BD Biosciences	1:25
CD14	MϕP9	Mouse IgG2b, κ	PERCP	345786	BD Biosciences	1:25
CD45	HI30	Mouse IgG1, κ	PERCP	304026	BioLegend	1:25
CD45	5B1	Mouse IgG1, κ	FITC	130-080-202	Miltenyi Biotec	1:25
CD45RO	UCHL1	Mouse IgG2a, κ	APC	340438	BD Biosciences	1:25
CD163	GHI/6	Mouse IgG1, κ	PE	556018	BD Biosciences	1:14,3 (1,75uL in 25uL)
MerTK	590H11G1E3	Mouse IgG1, κ	PECy7	367609	BioLegend	1:25
CD86	2331 (FUN-1)	Mouse IgG1, κ	PECy7	561128	BD Biosciences	1:25
HLA-DR	G46-6	Mouse IgG2a, κ	FITC	555811	BD Biosciences	1:33,33 (0,75uL in 25uL)
HLA-DR	G46-6	Mouse IgG2a, κ	PECy7	335830	BD Biosciences	1:33
CD11c	B-ly6	Mouse IgG1, κ	FITC	561355	BD Biosciences	1:25
CD11c	S-HCL-3	Mouse IgG2b, κ	PE	333149	BD Biosciences	1:25
CD11c	B-ly6	Mouse IgG1, κ	APC	559877	BD Biosciences	1:25
PD-L1	MIH1	Mouse IgG1, κ	PECy7	558017	BD Biosciences	1:20 (1,25uL in 25uL)
PD-L1	29E.2A3	Mouse IgG2b, κ	APC	329708	BioLegend	1:20
CD206	15-2	Mouse IgG1, κ	APC	321109	BioLegend	1:16,7 (1.5uLin 25uL)
CD206	19.2	Mouse IgG1, κ	FITC	551135	BD Biosciences	1:16,7 (1.5uLin 25uL)
CD3	SK7	Mouse IgG1, κ	BV421	563798	BD Biosciences	1:25
CD4	RPA-T4	Mouse IgG1, κ	APCH7	560158	BD Biosciences	1:25
CD8	SK1	Mouse IgG1, κ	PECy7	344712	BioLegend	1:25
CD20	L27	Mouse IgG1, κ	FITC	345792	BD Biosciences	1:25
CD25	M-A251	Mouse IgG1, κ	PE	555432	BD Biosciences	1:33,33
IL-6	MQ2-13A5	Rat IgG1, κ	FITC	501103	BioLegend	1:25 intra
S100A9	MRP 1H9	Mouse IgG1, κ	FITC	350703	BioLegend	1:10 (2,5uL in 25uL) intra
Ki67	B56	Mouse IgG1, κ	PE	556027	BD Biosciences	1:25 intra

Note: The table reports the lowest dilution used for the flow cytometry antibodies listed. Dilutions should be tested and adapted based on cell type and cell number.

Supplementary Table 2. Chromogenic IHC antibody details

Marker	Shown in Figure	Detection dye	Antigen Retrieval	Dilution	Supplier	Cat#	Isotype	Clone
CD31	Sup. Fig. 1	DAB	EDTA 10’	1/80	DAKO	M0823	Mouse IgG1,κ	JC70A
Collagen IV	Sup. Fig. 1	NovaRed	Pronase 6'	1/250	Sigma	C1926	Mouse IgG1,κ	COL-94
HLA-ABC	Fig 8, Sup. Fig. 12	NovaRed	EDTA 10’	1/100	Abcam	ab70328	Mouse IgG1,κ	EMR8-5
HMB45	Fig 8, Sup. Fig. 12	NovaRed	EDTA 10’	1/5	Immunologic	VWRKILM576211	Mouse IgG1,κ	HMB45
Tyrosinase	Fig 8, Sup. Fig. 12	NovaRed	EDTA 10’	1/20	Monosan	MONX10590	Mouse IgG2a	T311
MelanA	Fig 8, Sup. Fig. 12	NovaRed	EDTA 10’	1/100	Immunologic	VWRKILM022111	Mouse IgG1,κ	A103
SOX10	Fig 8, Sup. Fig. 12	NovaRed	EDTA 10’	1/100	Cell Marque	38R-16	Rabbit IgG	EP268
Ki67	Fig 8, Sup. Fig. 12	NovaRed	EDTA 10’	1/25	DAKO	m724001-2	Mouse IgG1,κ	MIB-1

Supplementary Table 3. Fluorescent IHC antibody details

Marker	Shown in Figure	TSA used	Antigen Retrieval	Dilution	Supplier	Cat#	Isotype	Clone
SOX10	Fig. 1, 3, 8 Sup. Fig. 3, 4	OPAL650	EDTA 10’	1/5000	Cell Marque	38R-16	Rabbit IgG	EP268
Tyrosinase	Fig. 1, 3, 8 Sup. Fig. 3, 4	OPAL650	EDTA 10’	1/200	Monosan	MONX10590	Mouse IgG2a	T311
Ki67	Fig. 1	OPAL570	EDTA 10’	1/25	DAKO	m724001-2	Mouse IgG1,κ	mib-1
FSP1	Fig 1, Sup. Fig. 3	OPAL570	EDTA 10’	1/2000	Sigma Aldrich	AMAB90599	Mouse IgG1,κ	CL0240
Cleaved Caspase-3	Fig. 2, Sup. Fig. 4	OPAL520	EDTA 10’	1/2000	Cell Signaling	9664	Rabbit IgG	5A1E
CD45	Fig. 2, 3, Sup. Fig. 4	OPAL570	EDTA 10’	1/750	DAKO	M0701	Mouse IgG1,κ	2B11+ PD7/26
FAP	Fig. 3, Sup. Fig. 3	OPAL690	EDTA 10’	1/100	Abcam	ab227703	Rabbit IgG	SP325
HMB45	Fig. 8	OPAL650	EDTA 10’	1/100	Immunologic	VWRKILM576211	Mouse IgG1,κ	HMB45
MelanA	Fig. 8	OPAL650	EDTA 10’	1/300	Immunologic	VWRKILM022111	Mouse IgG1,κ	A103
CD3	Fig. 8	OPAL520	EDTA 10’	1/400	Thermo Scientific	RM9107	Rabbit IgG	sp7
CD8	Fig. 8	OPAL690	EDTA 10’	1/1600	DAKO	M7103	Mouse IgG1,κ	C8/144B

Supplementary Table 4. Oligonucleotides Used for Quantitative RT-PCR

F: Forward primer. R: reverse primer.

Genes	Primer Sequence (5'-3')
IL6	F: GACAGCCACTCACCTCTTCAGAACG<br>R: ATCCATCTTTTTCAGCCATCTTTGG
XBP1	F: CCCTCCAGAACATCTCCCCAT<br>R: ACATGACTGGGTCCAAGTTGT
THBS1	F: TGCTATCACAACGGAGTTCAGT<br>R: GCAGGACACCTTTTTGCAGATG
TLR8	F: GGTCCTCTGCTCAGGGTGTCT<br>R: TGAATCCAGAAAACAACCACATG
PTGS2	F: GAATCATTCACCAGGCAAATTG<br>R: CTGTACTGCGGGTGGAACATT
TLR4	F: GGTGAGTAATTCCATGGTGCACTA<br>R: TTCCCTCTGCACTGGAAGCT
SPP1	F: GATAGTGTGGTTTATGGACTGAG<br>R: TTGTATGCACCATTCAACTCC
IDO1	F: TCTCATTTCGTGATGGAGACTGC<br>R: GTGTCCCGTTCTTGCATTTGC
GZMB	F: TGGGGGACCCAGAGATTAAAA<br>R: TTTCGTCCATAGGAGACAATGC
HIF1A	F: CACCACAGGACAGTACAGGAT<br>R: CGTGCTGAATAATACCACTCACA
ACTB	F: CTGGAACGGTGAAGGTGACA<br>R: AAGGGACTTCCTGTAACAACGCA

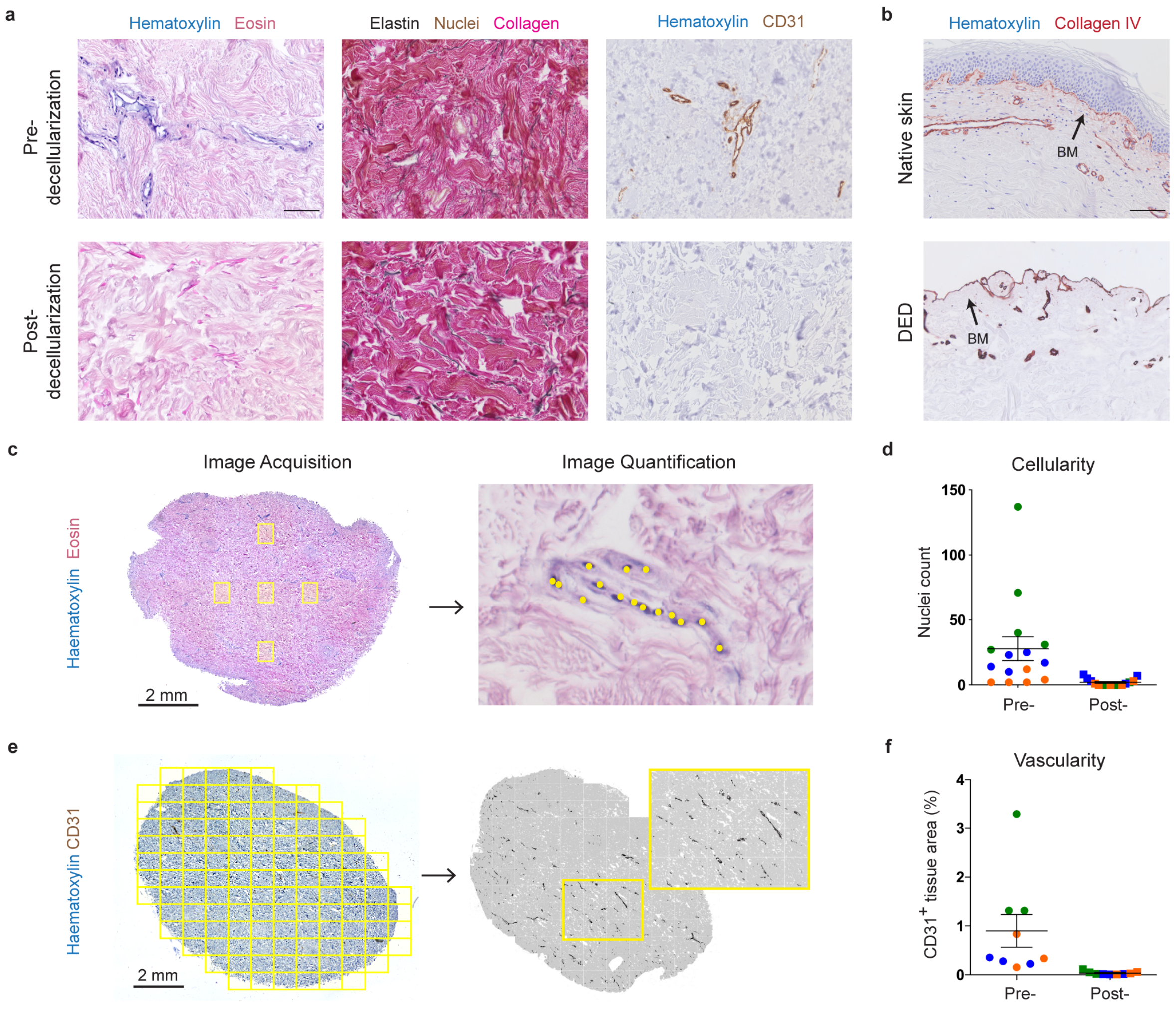

Supplementary Figure 1. Skin biopsy decellularization results in cell- and vessel-free de-epidermized human dermis, with a preserved structural matrix and basal layer.

a, Representative IHC evaluation of cellular (haematoxylin-eosin), extracellular matrix (Elastica van Gieson) and endothelial (CD31) components in pre- and post-decellularization skin samples (n=3). **b,** Representative staining of basement membrane (BM, Collagen type IV) in native skin and de-epidermized human dermis (n=3). All images (**a,b**) have the same magnification, scale bar 100µm. **c,d** Comparison of the quantitative assessment of dermal cell densities before and after decellularization. Nuclei (haematoxylin) were evaluated in 5 predefined areas (20x images) by independent histopathologic reviews using a digital imaging software. Dot plots of haematoxylin^{+} nuclei enumeration (**d**, "cellularity"); matched-colour symbols indicate three different donors, with n=5 replicates per donor. **e,f** Extent of endothelial cells in pre- *versus* post-decellularization samples was determined as the percentage of CD31^{+} signal over the whole tissue area. Matched-colour dots in graph (**f**, "vascularity") show replicate measurements of three distinct donors, with n=3 replicates per donor. Dot plots are Mean±SEM. Source data are provided as a Source Data file.

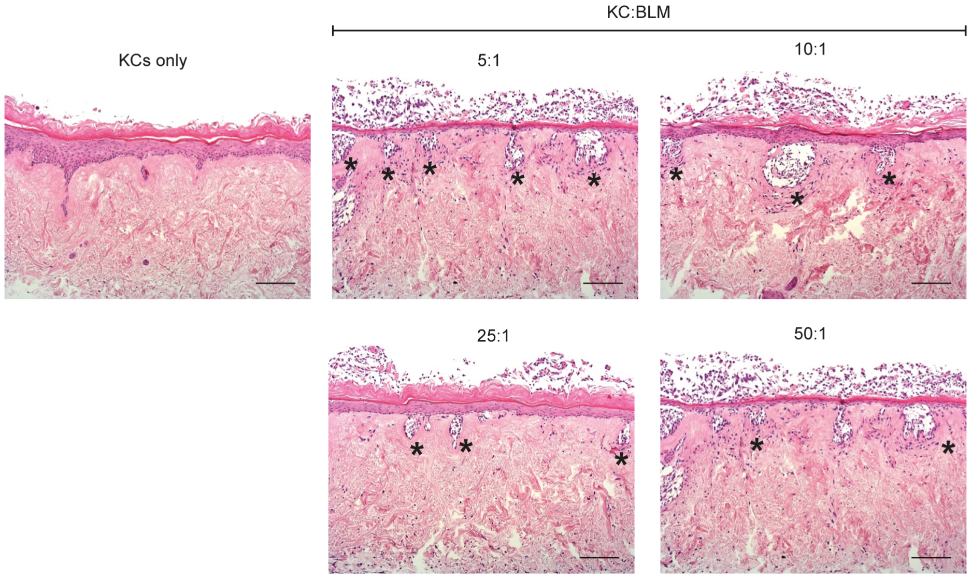

Supplementary Figure 2. Melanoma growth in human organotypic cultures can affect epidermal morphology.

Haematoxylin-eosin staining showing how epidermal morphology is affected by high melanoma cell density. Different keratinocytes (KCs) and BLM melanoma cell ratios were co-seeded onto a de-epidermized human dermis to find the optimal KC:BLM ratio, which ensures complete KC growth and differentiation into a fully-developed epidermis. Optimal ratio (25:1) was determined in n=1 experiment, and confirmed by HE staining in all subsequent experiments performed and reported in this manuscript. Interspersed tumour clusters are indicated with an asterisk. Scale bar 100µm.

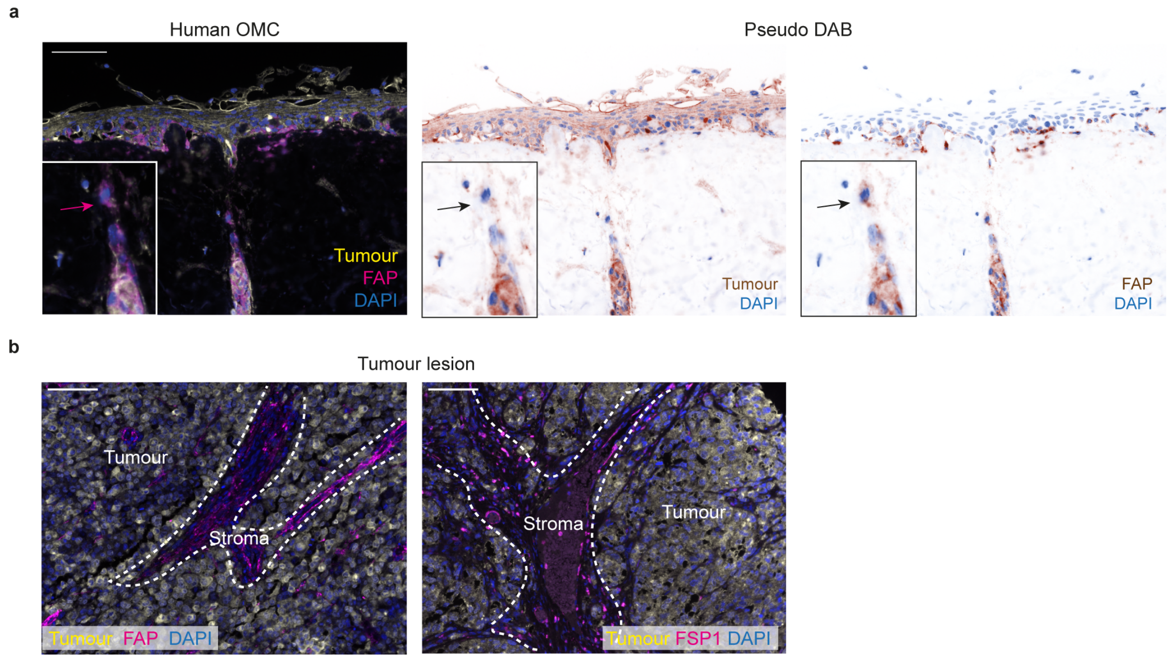

Supplementary Figure 3. IHC visualization of fibroblasts in OMCs and tumour lesions

a, Multiplex fluorescence IHC shows representative area (n=3) of the OMC containing fibroblasts (Fibroblast-Associated Protein, FAP^{+} cells, magenta) and melanoma cells (Tumour marker (tyrosinase and SOX10)$^{+}$ cells, yellow). DAPI (blue) indicates nuclei. Inserts show area at higher magnification. Middle and right images show the "Pseudo-DAB" of isolated fluorescent channels, of tumour and FAP respectively, of the same areas and fibroblasts are indicated with arrows. Some unspecific staining for Tumour marker is detected in the epidermis (light yellow). Scale bars 100µm. **b**, IHC image of triple FAP/Tumour marker/DAPI staining (left) and FSP-1/Tumour marker/DAPI staining (right) on control melanoma lesion (n=1). Scale bars 100µm.

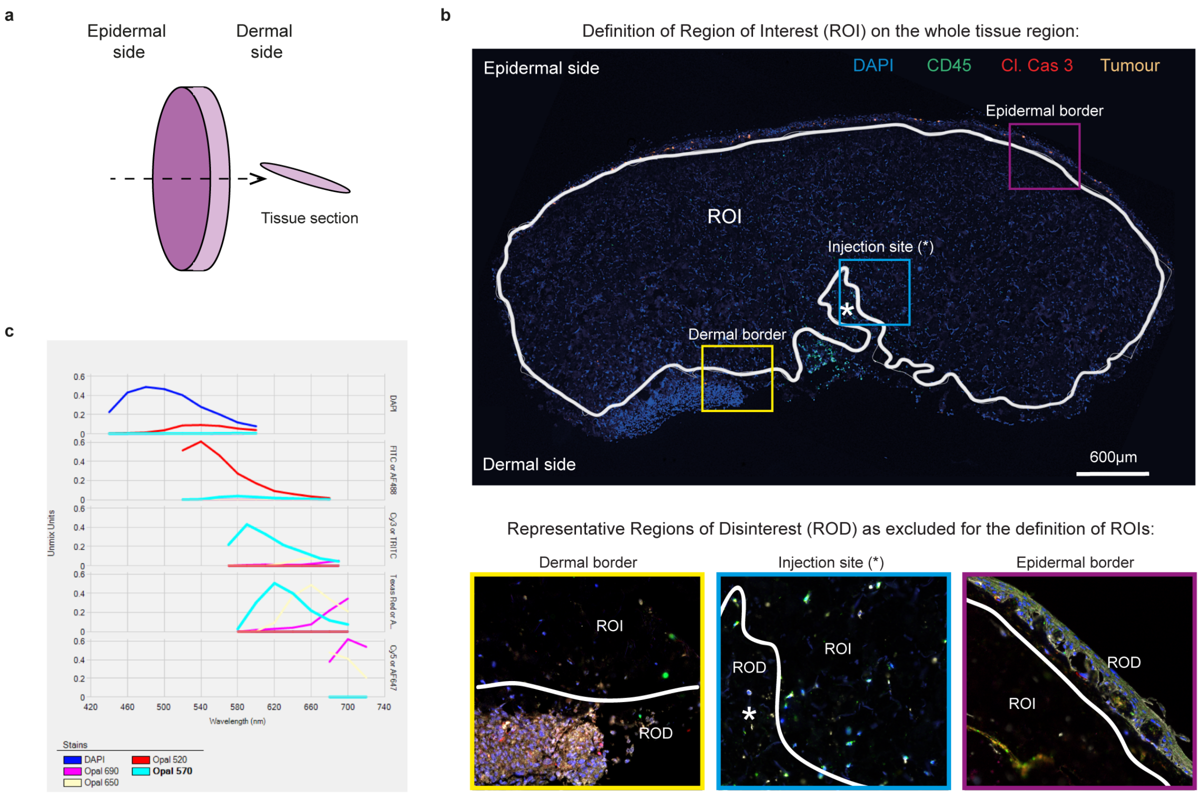

Supplementary Figure 4. Analysis of OMC sections by means of fluorescent multispectral imaging
a, Schematic representation of tissue sectioning for the analysis of immune cell distribution. **b,** Definition of the Region Of Interest (ROI) on the unmixed fluorescent IHC image containing DAPI (blue), CD45 (green), cleaved caspase 3 (Cl. Cas3, red), Tumour (yellow), used to perform qualitative and quantitative analysis in inForm (n=2). Examples of Regions of Disinterest (ROD) excluded from the analysis are shown: dermal border including tumour cluster (left), immune cell injection site (middle), epidermal border showing unspecific positivity for Tumour marker (right). **c,** Spectral library from multispectral IHC representative image. Individual examples of Cl. Cas3 (Opal520), CD45 (Opal570), Tumour (Opal650), FAP (Opal690), and DAPI, as well as autofluorescence from an unstained section, were spectrally analysed to generate a spectral library in support of multispectral unmixing in a multiplexed assay.

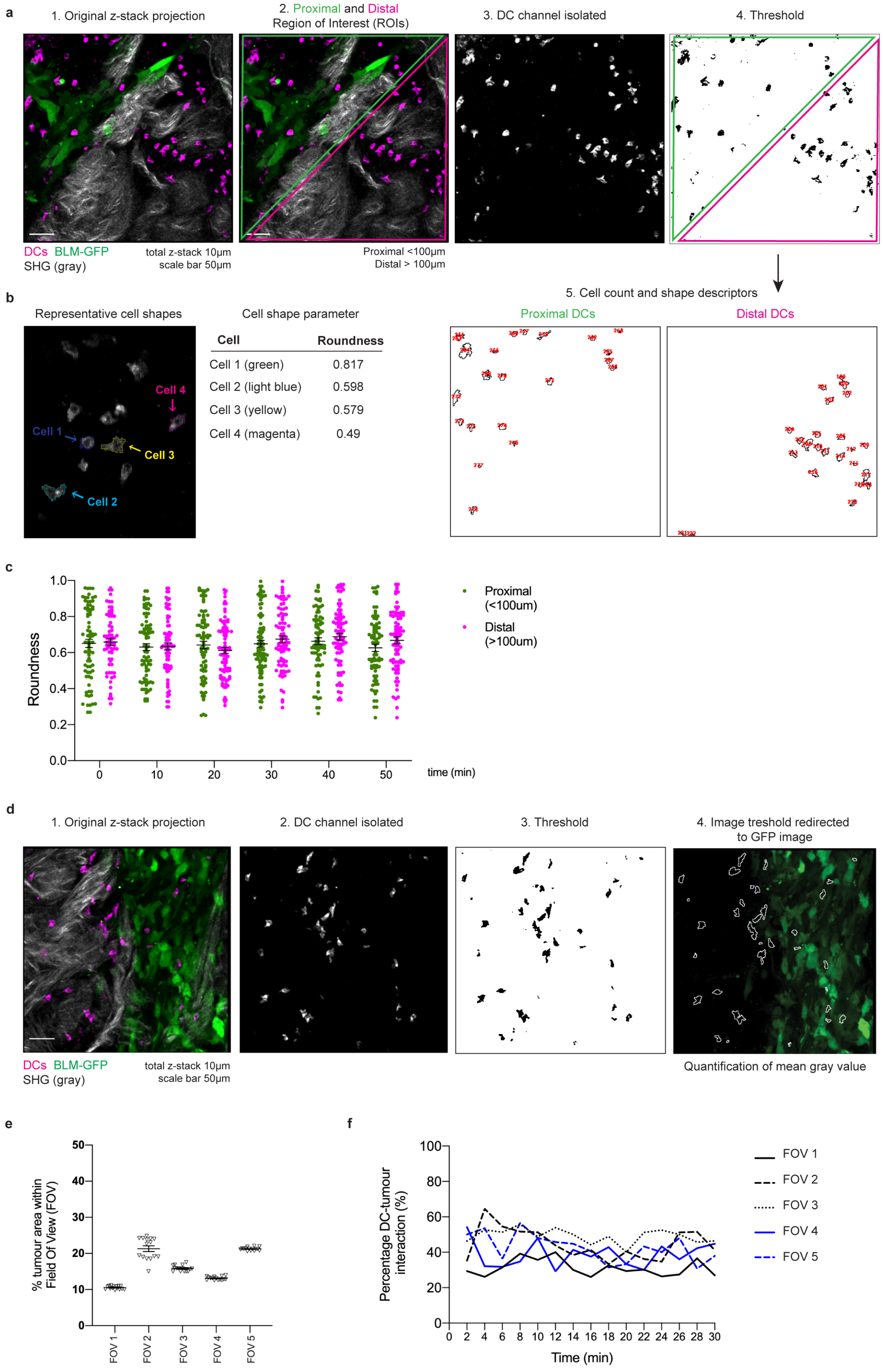

Cell	Roundness
Cell 1 (green)	0.817
Cell 2 (light blue)	0.598
Cell 3 (yellow)	0.579
Cell 4 (magenta)	0.49

Supplementary Figure 5. 2-photon imaging analysis of cell morphology of DCs and their interaction with tumour cells

a, Step-by-step imaging analysis of DC shape with respect to their distance from tumour area. 1, representative two-photon microscopy image of OMC containing DCs (magenta), tumour cells (green) and collagen fibers (second harmonic generation, SHG). The representative image is a maximum intensity projection of 3 consecutive z-stacks, for a total 10µm z-size (step size 5µm). 2-5, output images of semi-automated imaging process: 2, regions-of-interest (ROIs) defining *Proximal* (<100µm from tumour) and *Distal* (>100µm from tumour) areas. 3, channel image containing DCs; 4, overlay of threshold image of DC channel with *Proximal* and *Distal* ROIs; 4, DC count and analysis of cell shape within *Proximal* and *Distal* ROIs. N=2, whereby at least 2 different areas were imaged over time. **b,** Representative cellular shapes and their respective roundness values. **c,** Analysis of roundness factors of DCs over time, with respect to their proximity to tumour. Cell clusters were excluded from the analysis based on size. Total imaging time 50min. Dot plots are Mean±SEM. Statistics (all p values > 0.05) was calculated using two-way mixed ANOVA. **d,** Step-by-step imaging analysis of DC-tumour interactions. 1, representative two-photon microscopy image of OMC containing DCs (magenta), tumour cells (green) and collagen fibers (second harmonic generation, SHG). The representative image is a maximum intensity projection of 3 consecutive z-stacks, for a total z-size of 10µm (step size 5µm). 2-4, output images of semi-automated imaging process: 2, channel image containing DCs; 3, threshold image of DC channel; 4, overlay of resulting mask with the original tumour image, used to measure GFP expression (as mean gray values of pixel intensities) within DC selections. N=2, whereby at least 2 different areas per OMC were imaged over time. **e,** Percentage of area covered by tumour cells, within the five fields-of-view (FOVs) used for the analysis. Dot plots are Mean±SEM. **f,** The percentage of DCs interacting with tumour cells in a given time is comprised between 20% and 60%, over the total DCs present in a FOV. Total imaging time is 30min, at 2-min intervals. Source data are provided as a Source Data file. Scale bars 50um.

a

b

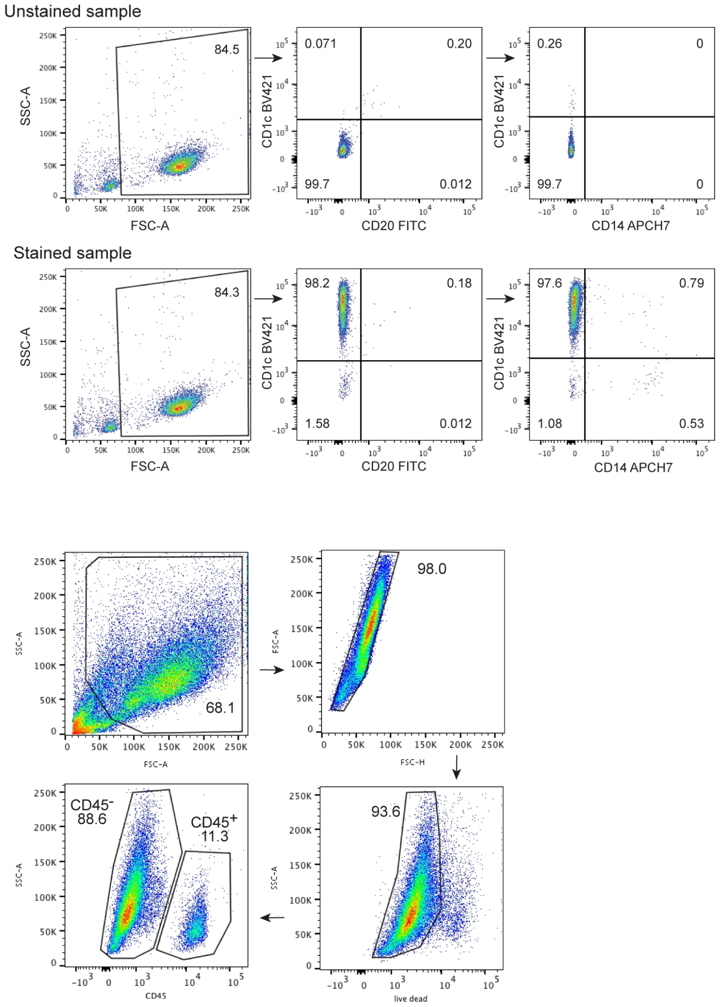

Supplementary Figure 6. Representative gating strategies for flow cytometry analysis of cellular phenotype

a, Purity of freshly isolated cDC2s from peripheral blood of healthy donors. Highly pure CD1c$^+$CD14$^-$ (> 96%) cells were obtained from healthy donor PBMCs through magnetic cell sorting (MACS) using the CD1c (BDCA1) DC isolation kit, combined with a pre-depletion step of monocytes using CD14-MACS microbeads. Cells were stained with primary directly-labelled antibodies: anti-CD1c, anti-CD14 and anti-CD20 Abs and the purity was assessed by flow cytometry. Representative dot plots of unstained and stained samples are shown. **b,** Representative gating strategy for the definition of single, live immune CD45$^+$ and non-immune CD45$^-$ cells in digested OMCs.

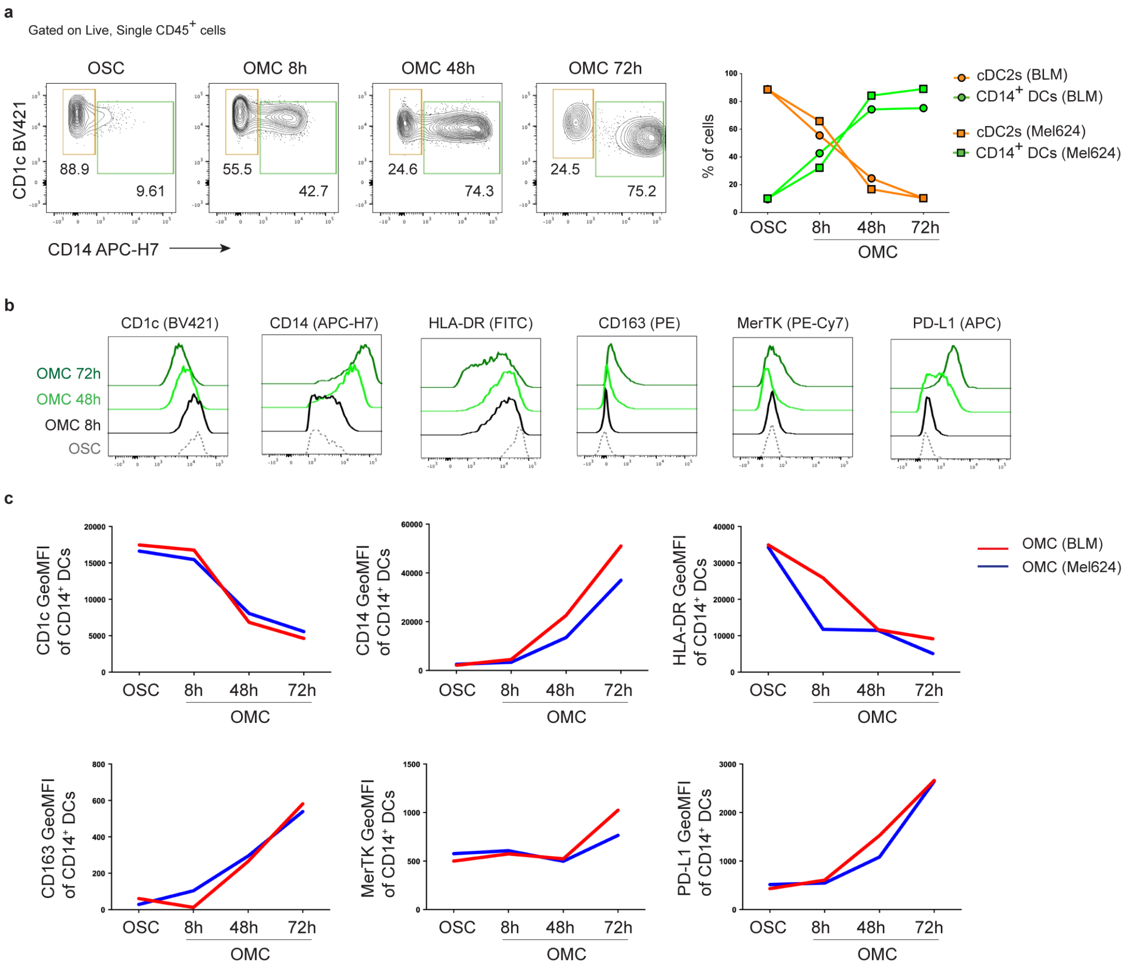

Supplementary Figure 7. Time course analysis of cDC2 phenotype in OMC

a, 8hours (h), 48h, 72h after cDC2s injection, OMCs were digested and cell suspensions were stained. Representative flow cytometry contour plots showing gated cDC2s ($CD1c^{+}CD14^{-}$ cells) and $CD14^{+}$ DCs ($CD1c^{+}CD14^{+}$ cells). Representative plots of OMCs with Mel624 melanoma cell line are shown. Numbers indicate the percentage of gated cells. Graph shows the frequency of cDC2s and $CD14^{+}$ DCs in OSC or OMC over time, generated either with BLM or Mel624 (n=2). **b**, Representative flow cytometry histograms for each indicated marker in OSC and OMC across different time-points. **c**, Geometric mean of the fluorescence intensity (GeoMFI) for the indicated marker in gated $CD14^{+}$DCs cultured within OSC or over-time in OMC, generated either with BLM or Mel624. Source data are provided as a Source Data file.

a

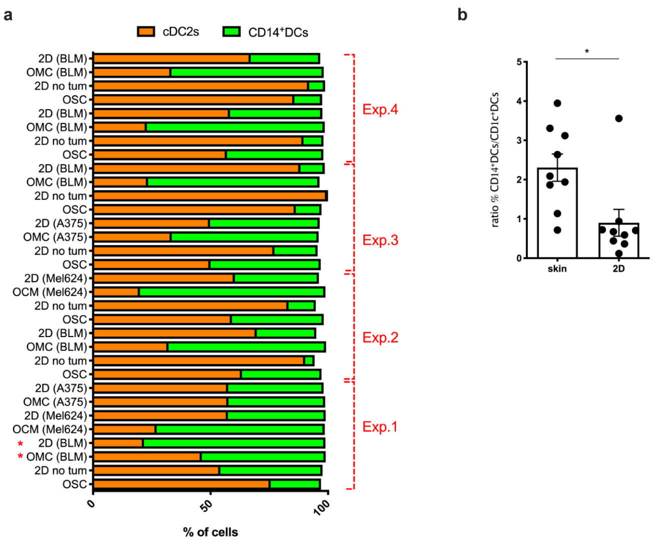

c

Exp 1	CD14+DCs/cDC2s BLM		CD14+DCs/cDC2ss A375		CD14+DCs/cDC2s Mel624	
	Skin	2D	Skin	2D	Skin	2D
HLA DR	0,2952	0,3770	0,2765	0,4273	0,4442	0,4238
CD163	1,6289	1,4040	1,6667	1,1957	3,6165	1,2159
PD-L1	1,9865	1,4603	1,6968	1,2828	1,7024	1,0776
MerTK	2,1831	0,7598	2,0262	1,3989	2,3315	1,3318

Exp 2	CD14+DCs/cDC2s BLM		CD14+DCs/cDC2s Mel624	
	Skin	2D	Skin	2D
HLA DR	0,3545	0,1170	0,5064	0,3351
CD163	2,2798	1,2999	1,9833	1,3665
PD-L1	1,4491	0,7340	1,9431	1,3286
MerTK	1,6237	1,0664	1,9350	1,4759

Exp 3	CD14+DCs/cDC2s A375	
	Skin	2D
HLA DR	0,6096	0,2636
CD163	2,0365	1,2424
PD-L1	0,9394	0,8145
MerTK	2,1339	1,2585

Exp 4	CD14+DCs/cDC2s BLM	
	Skin	2D
HLA DR	0,4270	0,2507
CD163	1,6271	0,9843
PD-L1	1,6636	0,6539
MerTK	0,7816	0,4727

low high

* "Skin" indicates organotypic cultures

Supplementary Figure 8. CD14+DC percentage and phenotype in conventional co-cultures (2D) and OMCs

Intra-donor comparison of cDC2s cultured with tumour cells (BLM, A375, Mel624) in conventional 2D co-cultures *versus* OMCs (n=9, over 4 experiments). **a,** Graph showing the percentages of cDC2s and CD14+DCs. Red asterisk indicates the only tumour condition (BLM) in which we detected a higher percentage of CD14+DCs compared to cDC2s, in 2D *versus* OMCs. **b,** Histograms indicate ratio of percentages of CD14+DCs and cDC2s (p=0.0109) in conventional 2D co-cultures *versus* OMCs (n=9; Mean±SEM, two-tailed unpaired t test). **c,** Heatmap summarizing ratio of GeoMFI's of CD14+DCs and cDC2s for the indicated markers (HLA-DR, CD163, PD-L1, MerTK). Red: higher expression in CD14+DCs, blue: lower expression in CD14+DCs. "Skin" indicates organotypic cultures. Source data are provided as a Source Data file.

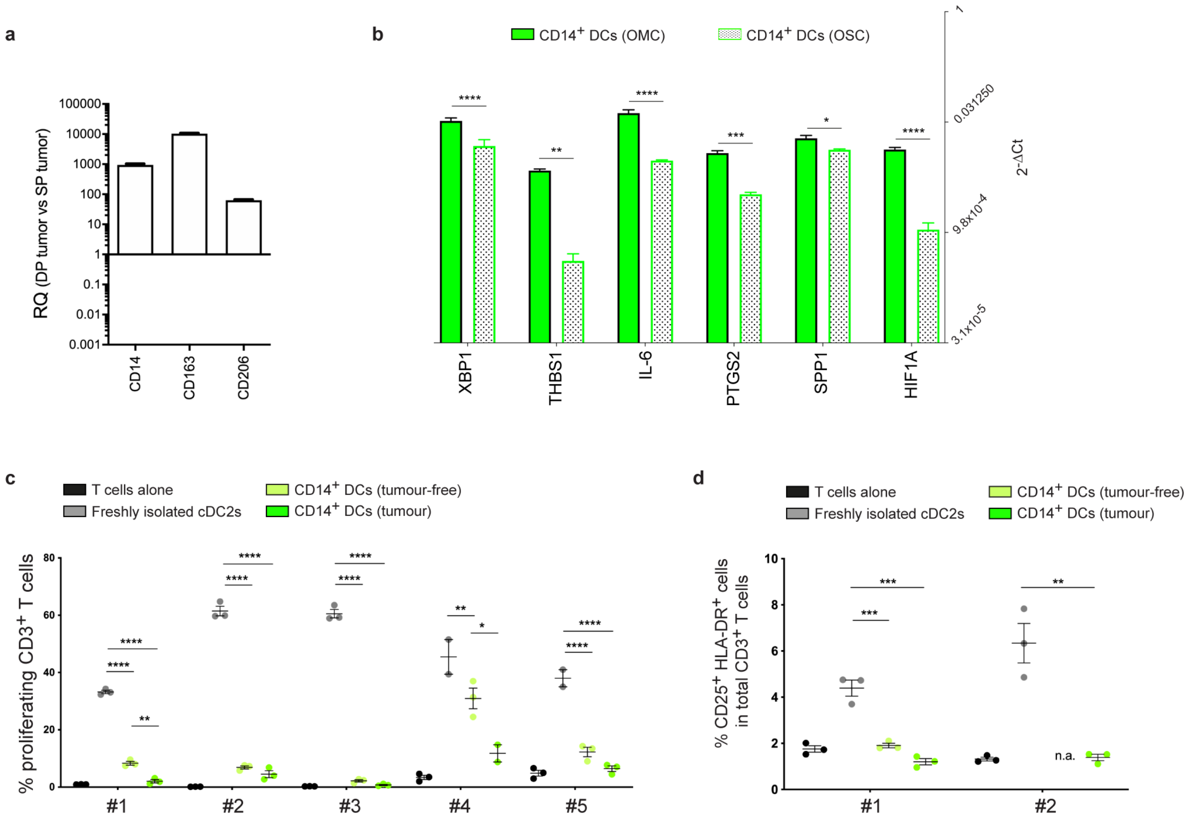

Supplementary Figure 9. Functional characterization of CD14^{+}DCs in OSCs and OMCs

a, b, 2 days after total cDC2s injection, OSCs and OMCs were digested and CD1c^{+}CD14^{+} (CD14^{+}DCs) subsets FAC-sorted for RNA extraction and molecular characterization by qRT-PCR. **a,** Graph reports the Relative Quantification (RQ) in CD14^{+}DCs (OMC) using cDC2s (OMC) as calibrator. **b,** Gene expression levels ($2^{-\Delta Ct}$) for the indicated genes in CD14^{+} DCs isolated from OSCs and OMCs are shown. ACTB was used as an internal reference. Reported values for each gene (n=3 biological replicates) are Mean±SEM; one-way analysis of variance (ANOVA), Sidak's multiple comparisons. **c,d** Proliferation of allogeneic (**c**) and activation of autologous (**d**) CD3^{+} T cells 5 days after co-culture with tumour-conditioned and tumour-free CD14^{+} DCs. T cells alone and cDC2s prior to tumour conditioning were used for comparison. **c**, Scattered dot plots (five independent experiments, with three replicates per condition (except for Exps#4 and #5, with at least 2 replicates; Mean±SEM; one-way analysis of variance (ANOVA) and Tukey's multiple comparisons tests). **d,** T cells were stained for HLA-DR, CD25, CD3, CD8 and live/dead marker. Percentage of activated CD25^{+}HLA-DR^{+} cells in total CD3^{+} T cells is reported. Scattered dot plots (two independent experiments, with three replicates per condition; except for Exp#2 - CD14^{+}DCs (OSC) for which not enough cells could be FAC-sorted for functional assay (n.a., not available). Mean±SEM; one-way analysis of variance (ANOVA) and Tukey's multiple comparisons tests). Statistical significance was annotated as follows: *p < 0.05, **p < 0.01, ***p < 0.001, ****p < 0.0001. Source data are provided as a Source Data file.

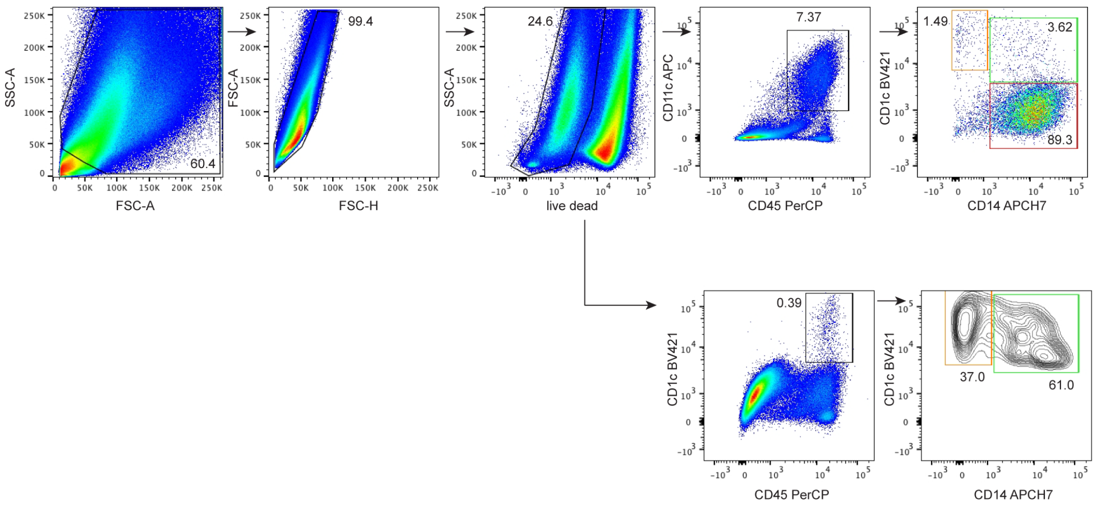

Supplementary Figure 10. Gating strategy applied for the analysis of the patient tumour suspension. Representative dot plots illustrating the gating strategy applied in the tumour suspension to identify: cDC2s (orange), $CD14^{+}$ DCs (green), and $CD14^{+}$ monocytes/macrophages (red) within total immune myeloid $CD45^{+}CD11c^{+}$ cells and cDC2s (orange), $CD14^{+}$ DCs (green) in gated $CD45^{+}CD1c^{+}$ cells.

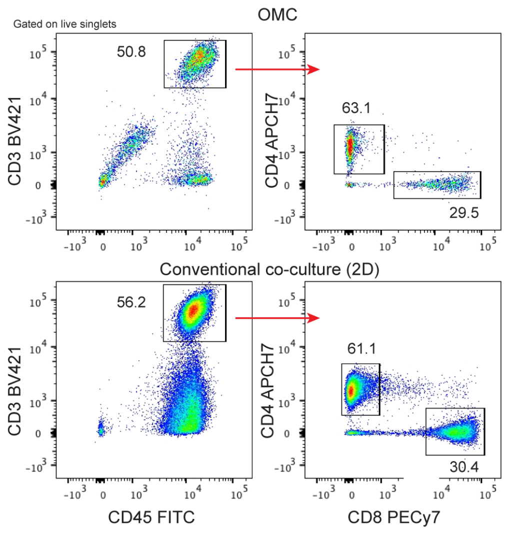

Population	% of parental population	
	OMC	Conventional co-culture (2D)
CD3⁺	50.8	56.2
CD4⁺	63.1	61.1
CD8⁺	29.5	30.4

Supplementary Figure 11. Culture of T lymphocytes and human melanoma cell suspension in human dermal scaffolds. Intra-donor comparison of PBMCs cultured for 2 days in the OMCs *versus* conventional 2D culture tubes. For analysis, PBMCs were stained with anti-CD45, anti-CD3, anti-CD4 and anti-CD8 antibodies. After excluding doublets and dead cells, $CD45^{+}CD3^{+}$ cells were sub-gated in $CD4^{+}$ and $CD8^{+}$ cells.

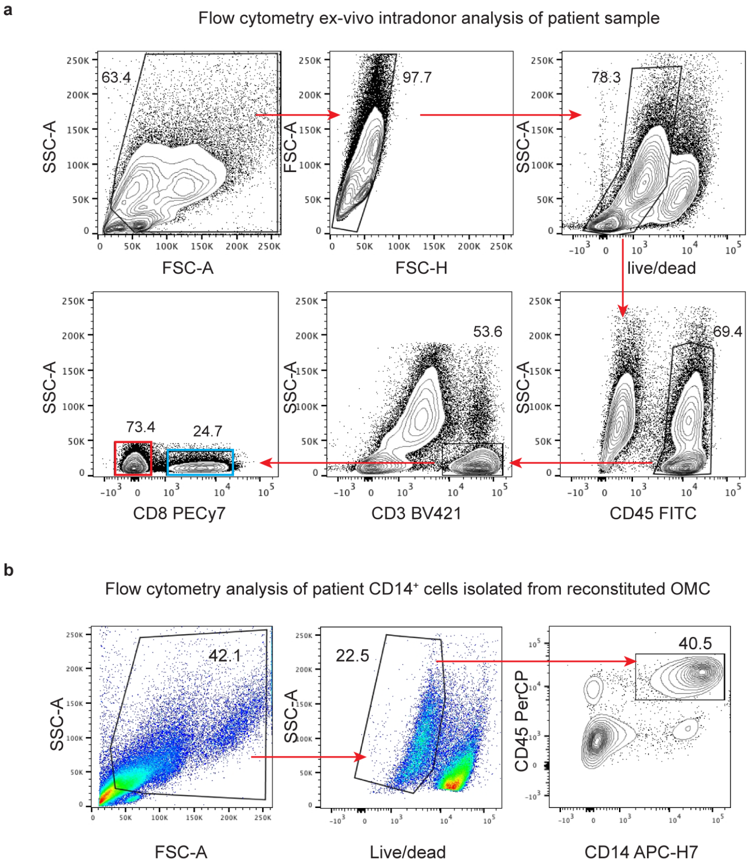

Supplementary Figure 12. Gating strategy applied for the *ex vivo* analysis on patient tumour suspension and CD14^{+} cells isolated from the reconstituted OMC. a, Prior to injection, melanoma patient's suspension was stained with anti-CD45, anti-CD3 and anti-CD8 antibodies. After excluding doublets and dead cells, CD45^{+}CD3^{+}CD8^{+} and CD45^{+}CD3^{+}CD8^{-} cells were quantified. **b,** Representative dot plots illustrating the gating strategy applied in the digested-OMC to identify CD14^{+} monocytes.

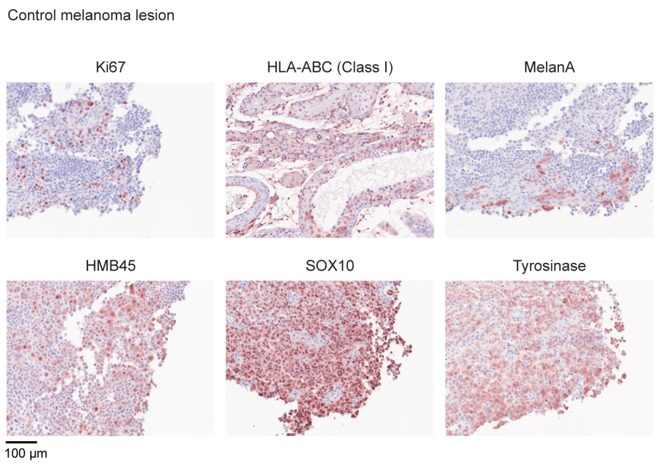

Supplementary Figure 13. IHC staining of tumour markers in control melanoma lesion

Representative IHC images of tumour-specific characteristics in control paraffin-embedded melanoma lesion. n=1 staining in control tissue was performed for each marker. Scale bar 100µm
