# Supplementary material for: The tumour microenvironment shapes dendritic cell plasticity in a human organotypic melanoma culture
Source: Nat Commun. 2020 Jun 2;11:2749. doi: 10.1038/s41467-020-16583-0 (PMC7265463; doi:10.1038/s41467-020-16583-0)
Supplement: Supplementary file 3 — Description of Additional Supplementary Files [file 41467_2020_16583_MOESM3_ESM.docx]

**Description of Supplementary Files**

**File Name: Supplementary Movie 1**

**Description:** Microinjection of immune cells into a dermal scaffold to generate OMCs. Step-by-step handling of dermal scaffols and injection of immune cells. Detailed methodology is described in the Methods section. 1, Remove transwell filter containing dermal scaffols; 2, Transfer dermal scaffold onto a petri dish (ensuring that the basal membrane side faces down); 3, Pipette cell suspension (previously prepared) into a 1ml-syringe attached to a microneedle array; 4- Gently inject cell suspension into the dermal scaffold; 5- insert OMC back into a transwell filter (basal membrane side down) in a 24well plate; 6, refresh culture media; 7, move transwell filter back into the well and add media on top of the OMC.Culture for an additional 24h-72h.

**File Name: Supplementary Movie 2**

**Description:** Two-photon imaging of cD2c DC sampling tumour fragment Two-photon time-lapse recording of cDC2s (PKH26, magenta)-tumour cell (GFP, green) interaction [λ (excitation) = 950nm]. Second harmonic generation (SHG, gray) shows collagen fibers of the extracellular dermal matrix. Duration of the video is 36min (18 frames at 2min interval) . Within the OMC, cDC2s actively interact with live tumour cells and sampled tumour-derived cellular microparticles. Representative time points are shown in Figure 3e. Scale bar 20µm.

**File Name: Supplementary Movie 3**

**Description:** Two-photon imaging of cD2c DC interacting with tumour cells Two-photon time-lapse recording of cDC2s (PKH26, magenta)-tumour cell (GFP, green) interaction [λ (excitation) = 950nm]. Second harmonic generation (SHG, gray) shows collagen fibers of the extracellular dermal matrix. Duration of the video is 32min (16 frames at 2min interval) . Within the OMC, cDC2s interact with tumour cells and blebs released by the BLM-GFP cells into the ECM. Representative time points are shown in Figure 3f. Scale bar 20µm.
