# Supplementary material for: The tumour microenvironment shapes dendritic cell plasticity in a human organotypic melanoma culture
Source: Nat Commun. 2020 Jun 2;11:2749. doi: 10.1038/s41467-020-16583-0 (PMC7265463; doi:10.1038/s41467-020-16583-0)
Supplement: Supplementary file 7 — Reporting Summary [file 41467_2020_16583_MOESM7_ESM.pdf]

# nature research

Corresponding author(s): Carl Figdor
Last updated by author(s): 25/04/2020

# Reporting Summary

Nature Research wishes to improve the reproducibility of the work that we publish. This form provides structure for consistency and transparency in reporting. For further information on Nature Research policies, see Authors & Referees and the Editorial Policy Checklist .

## Statistics

For all statistical analyses, confirm that the following items are present in the figure legend, table legend, main text, or Methods section.

| n/a | Confirmed | |
|---|---|---|
| ☐ | ☒ | The exact sample size (*n*) for each experimental group/condition, given as a discrete number and unit of measurement |
| ☐ | ☒ | A statement on whether measurements were taken from distinct samples or whether the same sample was measured repeatedly |
| ☐ | ☒ | The statistical test(s) used AND whether they are one- or two-sided<br>*Only common tests should be described solely by name; describe more complex techniques in the Methods section.* |
| ☒ | ☐ | A description of all covariates tested |
| ☐ | ☒ | A description of any assumptions or corrections, such as tests of normality and adjustment for multiple comparisons |
| ☐ | ☒ | A full description of the statistical parameters including central tendency (e.g. means) or other basic estimates (e.g. regression coefficient) AND variation (e.g. standard deviation) or associated estimates of uncertainty (e.g. confidence intervals) |
| ☐ | ☒ | For null hypothesis testing, the test statistic (e.g. *F*, *t*, *r*) with confidence intervals, effect sizes, degrees of freedom and *P* value noted<br>*Give P values as exact values whenever suitable.* |
| ☒ | ☐ | For Bayesian analysis, information on the choice of priors and Markov chain Monte Carlo settings |
| ☒ | ☐ | For hierarchical and complex designs, identification of the appropriate level for tests and full reporting of outcomes |
| ☒ | ☐ | Estimates of effect sizes (e.g. Cohen's *d*, Pearson's *r*), indicating how they were calculated |

*Our web collection on statistics for biologists contains articles on many of the points above.*

## Software and code

Policy information about availability of computer code

| | |
|---|---|
| Data collection | -Flow cytometry acquisition was performed with the FACSVerse (BD Bioscience) equipped with the FACS Suite software<br>-Gene expression data were collected with the CFX96 Bio-Rad.<br>-Whole tissue slides were imaged using Vectra Intelligent Slide Analysis System (Version 3.0.3, PerkinElmer Inc.)<br>-Phenochart (Version 1.0.9, PerkinElmer Inc.) was used to select Regions of interest (ROI) for analysis<br>-All time-lapse experiments were performed using a multiparameter multiphoton microscope (TriMScope-II, LaVision BioTec, Bielefeld, Germany). |
| Data analysis | -Flow Cytometry data were analyzed using FlowJo (Version 10, Treestar).<br>-Gene expression data were analyzed using the 2−ΔCt method, in which Ct represents the threshold cycle.<br>-Images of single stained tissues for each reagent were used to build spectral libraries of the single dyes by using the inForm Advanced Image Analysis Software (Version 2.4.1, PerkinElmer Inc.).<br>-Time-lapse images were analyzed using the open source-imaging platform, Fiji (imageJ 64 Bit for Windows).<br>-Heatmap showing concentrations of soluble factors among conditions were generated with Rstudio (Version 1.2.1335, RStudio Team 2018), using log2-transformed cytokine concentrations.<br>-Statistical analysis was performed using GraphPad Prism V6 and V8. |

For manuscripts utilizing custom algorithms or software that are central to the research but not yet described in published literature, software must be made available to editors/reviewers. We strongly encourage code deposition in a community repository (e.g. GitHub). See the Nature Research guidelines for submitting code & software for further information.

# Data

Policy information about availability of data

All manuscripts must include a data availability statement. This statement should provide the following information, where applicable:

- Accession codes, unique identifiers, or web links for publicly available datasets
- A list of figures that have associated raw data
- A description of any restrictions on data availability

All relevant data are available upon reasonable request from the corresponding authors. The source data underlying Figs 2d, 4b-e, 5a-d, 6a, 6d-f, 7b-c, 8b, 8e and Suppl. Figs 1d, 1f, 5c, 5e-f, 7a, 7c, 8a-b, 9a-d are provided as a Source Data file.

# Field-specific reporting

Please select the one below that is the best fit for your research. If you are not sure, read the appropriate sections before making your selection.

[x] Life sciences [ ] Behavioural & social sciences [ ] Ecological, evolutionary & environmental sciences

For a reference copy of the document with all sections, see nature.com/documents/nr-reporting-summary-flat.pdf

# Life sciences study design

All studies must disclose on these points even when the disclosure is negative.

| | |
|---|---|
| Sample size | -The sample size relative to in vitro experiments was determined according to previous experimental experience and was always ≥2.<br>-The sample size relative to the patient-derived material was not defined at priori, but determined by the availability of patient's blood and tumor specimen. |
| Data exclusions | No data was excluded |
| Replication | All in vitro experiments (both 3D and 2D) were repeated a minimum of two times and findings were consistent. Every figure states how many times each experiment has been repeated/how many replicates were performed. For all experiments involving melanoma patient-derived material, repetitions of distinct biological experiments was subjected to availability of the primary material. |
| Randomization | -For every 3D experiment: upon processing and characterization of each batch (=donor) to ensure complete decellularization, dermal scaffolds used to generate OMCs were randomly assigned to maintain inter-donor variability. Dermal scaffolds from the same batch (=donor) were always used within the same experiment.<br>-Upon isolation, cDC2s were tested for purity via flow cytometry. Only cDC2 cells yielding high purity (>95%) and recovery (=absolute number of isolated cells), suitable for the designed experiment, were used. |
| Blinding | -No blinding was applied for 2D and 3D in vitro experiment setup and analysis;<br>-For the histopathological evaluation of vascularity (CD31) and cellularity (nuclei counts) of dermal scaffolds, samples were randomized and blindly analyzed by two experienced pathologists, and using an image analysis software (either Inform or Fiji).<br>-Standard IHC (DAB or NovaRed) staining and image acquisition of patient-derived OMC were performed blindly by an experienced scientist. Corresponding patient-tumor lesions were collected, randomized and stained/analyzed blindly. Evaluation of the staining was carried out prior to accessing the Pathological Diagnostic Report, to confirm scoring of the tumour markers of interest.<br>-Multiplex fluorescent stainings on OMCs were not conducted blindly, while histological evaluation and cytometric image quantification were carried out blindly by a dedicated pathologist and bioinformatician, respectively.<br>-Multiplex fluorescent stainings on patient-derived OMC and multi-paramatric flow cytometry analysis on the same patient-derived tumor single cell suspension were performed blindly. |

# Reporting for specific materials, systems and methods

We require information from authors about some types of materials, experimental systems and methods used in many studies. Here, indicate whether each material, system or method listed is relevant to your study. If you are not sure if a list item applies to your research, read the appropriate section before selecting a response.

## Materials & experimental systems

| n/a | Involved in the study | |
|---|---|---|
| [ ] | [x] | Antibodies |
| [ ] | [x] | Eukaryotic cell lines |
| [x] | [ ] | Palaeontology |
| [x] | [ ] | Animals and other organisms |
| [ ] | [x] | Human research participants |
| [x] | [ ] | Clinical data |

## Methods

| n/a | Involved in the study | |
|---|---|---|
| [x] | [ ] | ChIP-seq |
| [ ] | [x] | Flow cytometry |
| [x] | [ ] | MRI-based neuroimaging |

## Antibodies

Antibodies used

All flow cytometry, chromogenic IHC antibodies and fluorescent IHC antibodies and related details are reported in Suppl Table1, Suppl Table 2 and Suppl Table 3, respectively. These tables already include Supplier, Catalog number, clone and dilution used.

Validation

All anti-human flow cytometry antibodies used in the study were validated by the commercial supplier. All validation statements can be found on the respective company website. In house titration was performed on positive control cells (PBMC, purified CD1c DCs) to define the best Stain Index for each antibody. We used fluorescence-minus-one (FMO) as negative control for each specific multi-parametric flow cytometry panel designed.

All IHC antibodies were titrated on positive control tissue, starting from the recommended dilution. Respective staining without primary antibodies were used as a negative control. To assess specificity we used not only melanoma tissue sections but also tissue microarrays (TMA) containing different types of tissue (e.g. lymph node, tonsil).

As previously stated in M. Gorris et al. Journal of Immunology 2017, in fluorescent multiplex IHC at equal Ab-concentration TSA-based quantification is expected to yield a higher positivity compared to standard IHC (DAB) staining. Definition of anti-CD45 Ab dilution to be used was defined on TMA, through serial dilution of the anti-CD45 ab to obtain staining levels and cell frequencies comparable to conventional DAB staining. Each Multiplex TSA was optimized as illustrated in M. Gorris et al. Journal of Immunology 2017.

A mix of monoclonal antibodies recognizing 4 melanoma antigens, HMB45 (gp100), MelanA (MART-1), Tyrosinase and SOX-10 was used to delineate melanoma within Patient-derived OMC and melanoma tissues. Alternatively, a selected cocktail of monoclonal antibodies directed towards only two melanoma-associated antigens anti-tyrosinase and anti-SOX10 was applied as Tumour cell marker in BLM-derived OMC, upon testing of each single antibodies/combinations on BLM agarcyto.

In order to phenotypically characterize fibroblasts within the OMC, we assessed the expression of different fibroblast-associated markers (Vimentin, aSMA, FAP and FSP1) by immunohistochemistry.

Vimentin #1: Clone (V9) Cat# NCL-L-VIM-V9 Novocastra 1:50 EDTA 10'
Vimentin #2: Clone (V9) Cat# AM074-5M Biogenex 1:4000 EDTA 10'
aSMA: Clone (1A4) Cat# a5228 Sigma Aldrich 1:4000 EDTA 10'
FAP#1: Polyclonal SAB4500839 Sigma Aldrich 1:100 EDTA 10'
FAP#2: Clone (SP325) ab227703 Abcam 1:100 EDTA 10'
FSP1: Clone (CL0240) AMAB90599 Sigma Aldrich 1:200 EDTA 10'

Firstly, we compared their staining patterns both in healthy human skin and in two different human melanoma tissue biopsies. Both Vimentin and aSMA expression were not restricted to fibroblasts, showing staining positivity in melanoma cells and other stromal cells (including pericytes), respectively. Furthermore, FAP (ab#1, Sigma Aldrich) showed strong non-specific staining at all concentrations tested, excluding issues that might be related to saturating antibody dilutions. Conversely, both FAP (ab#2, Abcam) and FSP1 antibodies specifically stained fibroblasts in both human skin and melanoma tissues. Secondly, we tested the suitability of the selected FAP (ab#2) and FSP1 antibodies to discriminate fibroblasts within the reconstructed OMC, and did not observe non-specific background staining on OMC tissue sections with either of the two antibodies. Together, we believe these observations support our choice for using both FAP and FSP1 for fibroblast identification in our system.

To set the cleaved-caspase 3 antibody, agarcytos from untreated (live) and oxaliplatin-treated BLM (already described to induced tumour cell apoptosis in Di Blasio S. et al, Oncoimmunology 2016) were prepared. Similarly to what described above, definition of the best Ab dilution to be used was defined through serial dilution of the ab to obtain staining levels and cell frequencies comparable to conventional DAB staining.

Optimized antibodies, buffers and procedures used to deterime positivity scores of Ki67 and HLA-ABC in tumor tissues were adopted from the Diagnostic Laboratory of the Department of Pathology, Radboud university medical center, Nijmegen, The Netherlands.

## Eukaryotic cell lines

Policy information about cell lines

Cell line source(s)

The following cell lines were used:
-Human melanoma cells (BLM, Mel624 and A375), Adult human dermal fibroblasts, were all obtained from ATCC.
-Mouse fibroblasts 3T3 cell line used to culture human isolated keratinocytes were available and used at the Department of Dermatology, Radboud university medical center, Nijmegen, The Netherlands.
-BLM-GFP were generated by Stefania Di Blasio. Please refer to Di Blasio S. et al, Oncoimmunology 2016.

Authentication

Short tandem repeat (STR) authentication profiling were obtained from ATCC (>99% identical)

Mycoplasma contamination

All cell lines were regularly tested negative for mycoplasma contamination.

| | |
|---|---|
| Commonly misidentified lines (See ICLAC register) | No misidentified cell lines used in the study. |

## Human research participants

Policy information about studies involving human research participants

| | |
|---|---|
| Population characteristics | The study includes fresh tumoral tissue and blood from 5 metastatic melanoma patients who underwent a surgical resection with palliative intent. The following cases were analysed: Patient 1, dermal and subcutaneous metastasis axilla; Patient 2, adrenal metastasis; Patient 3, liver metastasis; Patient 4, subcutaneous metastasis and Patient 5, brain metastasis (<br>After collection, fresh samples were immediately processed by the Department of Tumor Immunology to obtain PBMC and single cell tumor cell suspensions, as described in the methods section of the manuscript.<br>FFPE samples from the same patients were collected afterward from the Pathology Department for patient-derived OMC comparison. No sensitive or personal data were collected. |
| Recruitment | Metastatic melanoma patients were recruited within the study protocol, CMO dossier-number 2016-2758, approved by the Dutch Institutional Review Board. Relevant patients were approached and those who agreed to take part in this study and signed informed consent form were included. No self selection bias is anticipated. |
| Ethics oversight | Centrale Commissie Mensgebonden Onderzoek (CCMO) |

Note that full information on the approval of the study protocol must also be provided in the manuscript.

## Flow Cytometry

### Plots

Confirm that:

- [x] The axis labels state the marker and fluorochrome used (e.g. CD4-FITC).
- [x] The axis scales are clearly visible. Include numbers along axes only for bottom left plot of group (a 'group' is an analysis of identical markers).
- [x] All plots are contour plots with outliers or pseudocolor plots.
- [x] A numerical value for number of cells or percentage (with statistics) is provided.

### Methodology

| | |
|---|---|
| Sample preparation | An accurate description of the material source as well as of the staining procedures (including blocking step, incubation time and temperature) is provided in the material and methods section. |
| Instrument | FACSVerse (BD Biosciences) |
| Software | BD FACS Suite Software |
| Cell population abundance | Post-sorted samples were pure as check by reacquisition of the sorted sample. |
| Gating strategy | Suppl Figures 6, 10, 11 and 12 report the applied flow cytometry gating strategies |

- [x] Tick this box to confirm that a figure exemplifying the gating strategy is provided in the Supplementary Information.
